# Phage Therapy with a Focus on the Human Microbiota

**DOI:** 10.3390/antibiotics8030131

**Published:** 2019-08-27

**Authors:** Sharita Divya Ganeshan, Zeinab Hosseinidoust

**Affiliations:** 1 School of Biomedical Engineering, McMaster University, Hamilton, ON L8S 4K1, Canada; 2 Department of Chemical Engineering, McMaster University, Hamilton, ON L8S 4L7, Canada; 3 Farncombe Family Digestive Health Research Institute, McMaster University, Hamilton, ON L8S 4K1, Canada; 4 Michael DeGroote Institute for Infectious Disease Research, McMaster University, Hamilton, ON L8S 4L8, Canada

**Keywords:** microbiome therapy, phage therapy, evolution, antibiotic resistance

## Abstract

Bacteriophages are viruses that infect bacteria. After their discovery in the early 1900s, bacteriophages were a primary cure against infectious disease for almost 25 years, before being completely overshadowed by antibiotics. With the rise of antibiotic resistance, bacteriophages are being explored again for their antibacterial activity. One of the critical apprehensions regarding bacteriophage therapy, however, is the possibility of genome evolution, development of phage resistance, and subsequent perturbations to our microbiota. Through this review, we set out to explore the principles supporting the use of bacteriophages as a therapeutic agent, discuss the human gut microbiome in relation to the utilization of phage therapy, and the co-evolutionary arms race between host bacteria and phage in the context of the human microbiota.

## 1. Introduction

Bacteriophages (phages) are bacteria’s natural predators and could be employed for treating infections. Phage therapy was actively utilized immediately after discovery of phages in the early 1900s [1], years prior to the introduction of antibiotics in the 1940s, when infectious diseases were the leading cause of mortality and morbidity within human populations [2,3]. However, a lack of understanding of phage biology in the early days, combined with exaggerated claims, a lack of controlled trials, and poor documentation, among others, led to phage therapy being overshadowed by antibiotics in Western medicine [4]. Antibiotics have since been highly attractive due to their broad-spectrum activity, allowing them to be used against a wide range of infections without necessarily identifying or characterizing the exact infective agent(s).

With increased understanding of the role of human microbiota in our overall well-being, this broad-spectrum killing activity is increasingly presenting itself as a major disadvantage. We now know that a symbiotic relationship is established directly between the human gut microbiome (the population of bacteria, viruses, and fungi that occupy surfaces of the human body) and the physical and mental health of the human host, the disturbance of which could lead to the initiation or progression of many chronic and degenerative diseases [5,6,7,8,9]. The same is true for other niche microbiota such as nasal [10], eye [11], oral [12], and genital tracts [13]. As bacteria’s natural predators, bacteriophages offer a powerful advantage over antibiotics, namely that they can be highly specific, targeting only their host bacteria, suggesting a milder therapy towards niche microbiota. However, one of the critical apprehensions concerning lytic phage therapy is the potential for perturbations of the niche microbiota as a result of the strong selective pressure exerted by lytic phages on their host communities [14,15,16].

In this review, we provide an overview of the general use of bacteriophages as therapeutic agents, then discuss the relationship between bacteriophage therapy and the microbiota in the context of the gut microbiota and possible effects of phage therapy on the human microbiota.

## 2. Bacteriophages as Therapeutic Agents

### 2.1. Phage Biology

**Figure 1:**
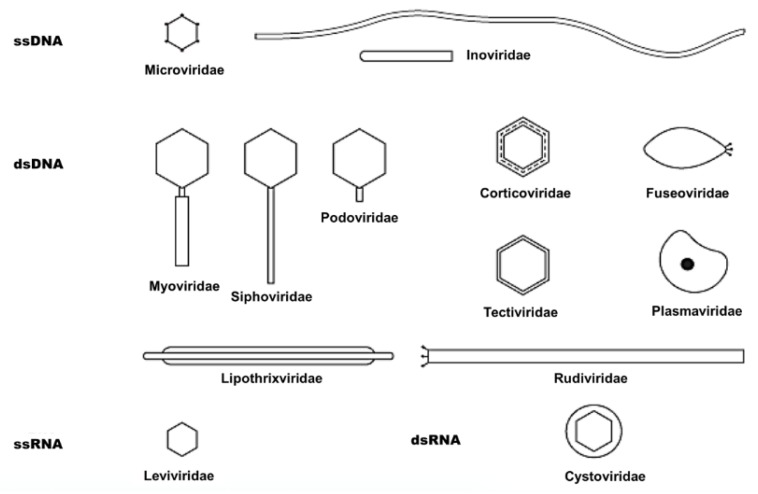
Schematic representation of major groups of bacteriophages. Reproduced with permission from reference [41].

**Figure 2:**
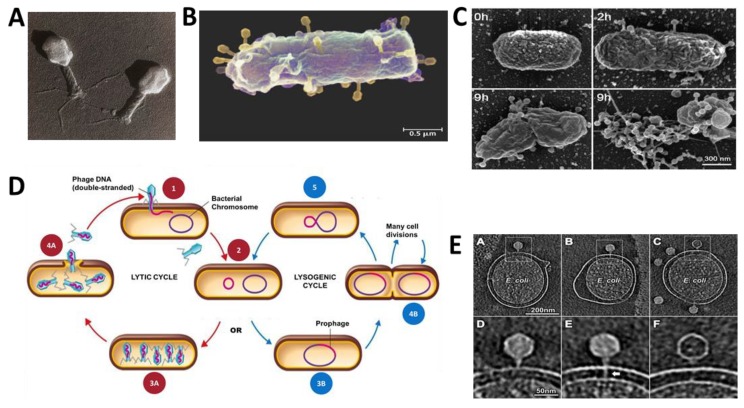
Introduction to lytic phage biology. (**A**) Shadowed Transition Electron Micrograph image of T4 phage (Mag 380,000×). This phage, a member in the *Myoviridae* family of the *Caudovirales* order, is one of the seven *Escherichia coli* phages (T1–T7) in this family. This image shows the icosahedral capsid head containing the genetic material, the contractile tail, and the long tail fibers of the phage. T4 head is approximately 90 nm, wide and the virion is 200 nm in length. This TEM was photographed at Wurtzbiozentrum at the University of Basel, reproduced with permission from references [42,43]. (**B**) Colorized scanning electron micrograph (SEM) images of multiple T4 bacteriophages infecting an *E. coli* cell reproduced with permission from reference [44]. (**C**) SEM images at different stages showing the infection of *Synechococcus* WH8102 by the S-TIM5 phage. 0 h-uninfected cells. 2 h-phage adsorption. 9-h cell lysis. 9-h viral release. These SEM images were collected from Sabehi. G., from the Israel Institute of Technology, reproduced with permission from reference [45]. (**D**) The lytic and lysogenic infection cycles. The first two stages are shared for both the cycles. Step 1- Attachment of the phage tail fibers to a specific receptor site on the bacterial cell wall and injection of the viral genome. Step 2- Phage DNA is then circularized and enters the lytic cycle or the lysogenic cycle. Lytic cycle: Step 3A- Synthesis of new viral proteins within the host. Step 4A- Virions are liberated as mature phages upon cell lysis. Lysogenic cycle: Step 3B- Phage DNA integrates within the bacterial chromosome by recombination, in turn becoming a prophage. Step 4B- Lysogenic bacterium reproduces normally and has the potential to do so over many cell divisions. The prophage may be released from the bacterial chromosome through external triggers, resulting in the initiation of the lytic cycle—reproduced with permission from reference [30]. (**E**) T7 bacteriophage infecting *E. coli* as seen with cryoelectron tomography at ~4 nm resolution. A/D- Adsorption of T7 phage into the outer membrane. B/E- Injection of the extended tail into the cell envelope. C/F- DNA ejection. These images are collected from Bo Hu, University of Texas—reproduced with permission from reference [31].

Bacteriophages (phages) are viruses of bacteria. With an estimated 10^32^ phages, these are the most ubiquitous and diversified biological group residing on Earth [17]. As a result of their obligate requirement of a bacterial host, bacteriophages are abundantly found distributed essentially anywhere their host exists in the biosphere [14,15,16], with ten to hundreds of millions of phages in every gram of soil [18], water [19], and billions on and inside the human body at any moment [20]. Bacteriophages are diverse in their complexity, structure, genetic material, and are variant in their shape (tailed, filamentous, and icosahedral), and size (Figure 1) [21]. Phage virions generally consist of a protein envelope (sometimes containing lipids) encapsulating a genome consisting of two to hundreds of kilobase pairs of single or double-stranded DNA or RNA [22]. Phage virions could be effectively visualized with electron microscopy; specifically, Transmission Electron Microscopy (TEM) (Figure 2A) [23].

Phages are more genetically diverse than their bacterial hosts (and prey); however, these bacterial viruses only infect a narrow range of bacteria that are closely related due to a combination of various factors. Such limiting factors include the specificity of the virion’s host binding proteins, biochemical interactions during infection, the presence of related prophages or particular plasmids in the host, and bacterial resistance mechanisms to phages (its predator) [24,25,26].

Bacteriophages are classified through a structural and sequence-based taxonomic system; initially into families, and each family is further categorized in accordance to the capsid structure, the structural and chemical composition of the genes and the mechanism of their mRNA production (Table 1) [27,28]. These viruses are further broadly categorized in terms of their propagation cycle as lytic, temperate, and chronic phages [29]. Regardless of the nature of their propagation cycle, bacteriophages first have to bind to specific sites on the host cell surface (Figure 2B). Lytic phages bind and adsorb to specific receptors on the host cell surface and inject their genome into the host cell and undergo propagation, which ultimately results in lysis of the host, further, releasing progeny phages into the surrounding medium (Figure 2C,D) [30]. This process is known as the lytic cycle. As an example, Figure 2E depicts the process of absorption and genome injection of the bacteriophage T7, a well-studied lytic bacteriophage belonging to the *Podoviridae* family [22]. T7, an *Enterobacteria* phage, attaches to outer membrane proteins OmpA and OmpF proteins on the bacterial cell wall, sending multiple internal capsid proteins into the host cell wall to construct an ejectosome from the tail of the phage and induce a pore within the bacterial cell wall. This then permits DNA from the capsid of the phage to translocate into the cell, and in turn, initiates the process to replicate phage DNA within the host [31]. Temperate viruses typically do not immediately “kill” the host bacteria; instead, they integrate their genome into the host chromosome, amplifying with every bacteria reproduction cycle; this embedded genome (known as a prophage) can be expelled from the genome of the host bacteria through the lytic cycle, once induced (Figure 2D) [30]. It is not clear what causes the induction of lytic cycle for temperate phages, but most factors that stress the host cell or cause DNA damage have been shown to induce temperate phages into a lytic cycle [32,33]. Thus, a temperate bacteriophage experiences both lytic and lysogenic cycles. This lytic-lysogeny switch has been a topic of research for decades [34,35]. In the lytic cycle, the phage replicates and lyses the host cell. In the lysogenic cycle, phage DNA is integrated into the host genome, where it is passed on to daughter cells. Phage λ is an example of a heavily studied temperate phage and uses the bacterial maltose pore LamB (λ-receptor) for delivery of its genome into the bacterial cell [36]. There is a third class of phages, known as the chronic phages, which do not lyse the cell or integrate their genome into the host genome, but instead use the host as a continuous phage-making factory—filamentous phage such as M13 belong to this class [29,37]. The common step in all three replication cycles is the specific binding of receptors on the phage virion surface to receptors on the bacterial host cell surface.

Phage therapy exploits the natural ability of bacteriophages to specifically target and kill host bacteria with high specificity, for the purpose of treating bacterial infections. For phage therapy, only strictly lytic phages are of immediate interest [38]. Although there are strong arguments to be made supporting the use of temperate phages for therapeutic purposes [39], regulatory agencies have a long way to go before accepting the use of viruses that could transfer their genes directly to bacterial cells in the body [40].

### 2.2. History of Phage Therapy

Frederick Twort, an English bacteriologist, first reported evidence of bacteriophages (lysis of bacterial cultures) in 1915 and suggested the effect might be due to the presence of a virus or an enzyme [46]. In 1917, Felix d’Herelle, a French–Canadian, and a self-taught microbiologist working at the Institute Pasteur, independently made similar observations, but he was quick to attribute the effect to a virus and named these microbes “bacteriophages” or bacteria-eaters; he was the first to utilize phages as antimicrobial agents to treat infections [47,48]. The discovery of phages played a crucial role in the development and understanding of further scientific discoveries, including the initiation of understanding the structure of DNA [49], and more recently, in the discovery of new clusters of regularly interspaced short palindromic repeat (CRISPR) systems [50].

Phage therapy was actively utilized, immediately by d’Herelle and later by others [1], for the treatment of infections years prior to the introduction of antibiotics in the 1940s, when infectious diseases were the leading cause of mortality and morbidity within human populations [2,3]. In 1919, d’Herelle treated three brothers, whose sister had just died of dysentery, with phages. The brothers, aged three, seven, and twelve were in a serious condition but began to recover in 24 h [4]. He then moved on to treating Bubonic plague in 1925 by injecting phages directly into the buboes, after which the patient showed signs of recovery in 24 h and exhibited full recovery in less than a month [51]. Data collected from the plague in India (in 1926) using phages isolated by d’Herelle showed a drastic decrease of mortality for the phage-treated group compared to the control group [51]. As the word spread, more trials followed, some of which were inconclusive. 

Antibiotics quickly overshadowed the use of phage therapy in Western medicine due to the lack of understanding of both phage biology and microbiology of infectious diseases, exaggerated claims, a lack of controlled trials, poor documentation, and politics [4]. This in turn, sparked scientific controversy about the treatment approach [52,53], rendering phage therapy essentially obsolete in Western medicine for close to four decades. Due to the occurrence of World War II, certain regions of the Soviet Union and Eastern Europe had limited access to antibiotics and thus focused on developing phage therapy [54]. The practice of phage therapy within the Soviet Union was well supported, and this is in fact still heavily practiced in Russia and the Eastern European countries for over 80 years, especially within the Eliava Institute in Tbilisi, Georgia, co-established by Felix d’Herelle himself [55,56].

### 2.3. Phage Therapy Compared to Antibiotic Therapy

As noted above, the 1940s brought upon a golden era for the utilization of antibiotics as antimicrobial agents. Penicillin was not immediately utilized as an antibiotic upon its serendipitous discovery in 1928 [3]. However, it was quickly guided into optimization due to the crucial necessity to treat sick or wounded soldiers in the US and Allies’ military forces during the war [57]. Antibiotics were discovered at a time when bacterial infections had a high mortality rate; antibiotics were highly attractive due to their broad-spectrum activity allowing them to be used against a wide range of bacteria without necessarily identifying or characterizing the exact infective bacterium. However, this main advantage was also a great disadvantage. Due to their relative non-specificity towards bacteria, antibiotics also destroy the commensal microflora, especially within the intestine for oral antibiotics. Similar effects on the microbiota are observed for other niches in the body. This is further associated with side-effects such as intestinal problems, or secondary infection, such as *C. difficile* infection [58]. Furthermore, secondary side-effects often occur as antibiotics are also mostly needed in repeated administrations [59]. Using lytic phage, however, could circumvent this issue because bacteriophages are very specific to their host. This means that phage therapeutics could be designed to kill an infection but not harm the microbiota. This specificity could, however, become a challenge because many infections are polymicrobial. This requires phage therapeutics to be cocktails of different phages rather than a pure stock [60].

The non-essential overuse and abuse of antibiotics (in clinics, aquaculture, and agriculture) has fueled the volatile era of antibiotic resistance globally. Specifically, a group of gram-positive and gram-negative species, made up of *Enterococcus faecium*, *Staphylococcus aureus*, *Klebsiella pneumoniae*, *Acinetobacter baumannii*, *Pseudomonas aeruginosa*, and *Enterobacter* species, known as ESKAPE pathogens, most of which are multidrug-resistant isolates, are the leading cause of hospital-acquired infections throughout the world. It is approximated that by the year 2050, we would have 10 million deaths per year, with more people dying from antibiotic resistance than cancer [61]. As a consequence of this current dangerous state of infective antibiotics and antibiotic resistance, interest in phage therapy as an antimicrobial strategy against lethal pathogens has resurfaced. A recent review investigating 30 clinical studies of phage therapy against ESKAPE pathogens, 87% showed efficacy and safety (67%) of the tested phages, but only 35% examined phage resistance [62].

## 3. Phage Therapy: Where Are We Now?

### 3.1. Advantages and Challenges of Phage Therapy

Bacteriophages are not a fully established alternative to antibiotics as an antibacterial therapeutic. However, they hold great promise not only for tackling antibiotic-resistant infections, but also for treating at-risk patients that are less tolerant to antibiotics such as infants, pregnant women, and immunocompromised patients. As phage therapy is in vogue right now as a potential alternative/adjuvant, it is imperative to carefully assess all possible characteristics, parameters, and limitations. Some advantages of phage therapy over antibiotics include:

- Specificity: Bacteriophages are generally very specific in their host range, which promises less harm to our microbiota. However, this specificity also means that for most clinically relevant infections, a mixture of different phage will have to be used to address the polymicrobial nature of the infection [60].
- Bactericidal versus bacteriostatic: lytic phages infect their target host bacteria and cause cell death, in comparison to certain bacteriostatic antibiotics [63,64].
- Active on-site propagation: bacteriophages increase in concentration in situ as they propagate in the presence of bacterial host (infectious agents). Unlike antibiotics, which often require frequent doses to kill the bacteria efficiently, only one bacteriophage is theoretically needed to target a single corresponding host bacterium (single-hit kinetics) [65]. It is possible to administer a single low-dose of phage, which will then propagate itself, given the existing bacterial density as an active therapy, resulting in continued bacterial adsorption and killing [66].
- Low inherent toxicity: bacteriophages are primarily composed of nucleic acid and proteins that have been studied to be non-toxic with the use of highly purified phage preparations [67].
- Enormous diversity: Phage is found in diverse abundances across the biosphere and therefore isolating and purifying new phages, necessary to target a known pathogenic bacteria, is achievable even when bacteria evolve resistance [68].
- Formulation and application versatility: Multiple strains of phages can be added together into a “phage cocktail” to target multiple bacteria of interest with a broader killing spectrum [69]. Furthermore, mode of administration (liquid, ointment [70], powder [71], oral tablets [72]) could also vary in accordance with each unique situation.
- Narrow potential for antibiotic cross-resistance: The mechanism of bacterial resistance to antibiotics and phage are entirely different [61,73]. Thus, bacteria that are resistant to antibiotics may be targeted and treated with the use of phage therapy, and vice-versa, presenting combination therapies as an attractive strategy [74].
- Biofilm clearance: phages have been demonstrated to lyse and penetrate through some biofilms that have shown resistance to antibiotics [75]. This is partially attributed to the presence of depolymerases and lysins that can chew through the biofilm extracellular polymeric matrix [76,77].
- Low environmental impact: bacteriophages are natural components of the environment that can be naturally evolved (as opposed to genetic modification), thus easing public acceptance of phage therapy. Furthermore, because this natural product is composed primarily of proteins and nucleic acids, unused phage materials can easily be inactivated and discarded [63].
- Relatively low discovery and production cost: the costs associated with the discovery, isolation, and purification of bacteriophages are significantly decreasing with the progression of screening and sequencing technologies [78,79].

### 3.2. Outstanding Challenges of Phage Therapy

Regardless of the numerous known advantages of bacteriophages, there remain outstanding challenges and un-addressed limitations to this approach that must be addressed and further investigated. In particular, there are currently four primary concerns:

- Narrow host-range: phages generally have a narrow host-range, which limits exactly which strain(s) of bacteria can be targeted. This is a challenge because most infections in the body are polymicrobial. Thus it is imperative to employ effective and efficient phage cocktails, curated from a combination of multiple selected phages to develop a broader host spectrum [80].
- Even though phage therapy was employed in Western medicine before antibiotics took over, it currently does not have the approval for human administration from the Food and Drug Administration (FDA) or the European Medicines Agency (EMA) due to an increase in regulatory standards, partially as a result of the increased understanding within the scientific community about the potential effect of drugs on the human microbiome and partially due to concerns about phage resistance and the role of phages in bacterial genome evolution. To obtain regulatory approvals, current research is heavily exploring roadblocks through numerous animal studies and clinical trials [81,82,83]. The FDA has, however, approved the use of phages for food decontamination [84], dietary supplements [85], and environmental prophylaxis. Phage is allowed only on a compassionate care basis for human therapeutic use [86,87].
- Phages as drivers of evolution: unlike common pharmaceuticals, bacteriophages are DNA/RNA-containing protein-based biological agents that have the potential to interact with the body’s immune system and other microbial cells in the body and can actively replicate and evolve within the body. This evolution can, in turn, result in the evolution of the commensal host bacterial communities and possibly even impact the composition of the niche microbiome [88].
- Phage selection criteria: The criteria for the selection of therapeutic phage is not well determined. Most reports to date have focused on phage host range, but factors such as the ability of phage to infect stationary phase bacteria [89], phage enzymes, stability to serum inactivation, and mutation rate have been shown to be important and deserve more investigation beyond proof-of-concept reports [68,90].
- Lack of well-curated public phage libraries: there is a serious lack of well-curated, well-characterized libraries for therapeutic phage. Various research labs in academia, industry, and army research centers have small to mid-sized collections that, except for very few cases, are considered proprietary and are not shared with the broader scientific community. This need was acknowledged by the community recently and led the DSMZ (German Collection of Microorganisms and Cell Cultures) to start the Phage Call Project and encourage researchers to deposit characterized phages to their public phage library. This effort, although valuable, is progressing very slowly, with only a few phages per strain currently in the library. The slow pace of progress in this regard can partially be attributed to the vast diversity of phages around the globe, partially to the lack of high throughput methods for rapid isolation and characterization of new phage, and partially to the lack of a mechanism to acknowledge phage as the intellectual property of the discovering lab, leading to the unwillingness of researchers to share so as to maintain competitive advantage.

### 3.3. Clinical Trials and Current Phage Therapy 

The Eliava Institute in Tbilisi Georgia remains a leading expert clinic for active practice of phage therapy, with hundreds of local, as well as international patients that have received lifesaving phage treatment [55]. Moreover, there have been several notable clinical trials of phage therapy within recent time. PhagoBurn, funded by the European Commission, was the first phage therapy clinical trial utilizing good manufacturing practices (GMP) [91]. Their phage cocktail was curated as a treatment for *Escherichia coli* and *Pseudomonas aeruginosa* burn wound infections. The development and validation of PhagoBurn served as an eye-opening experience for the community in terms of pitfalls in designing an efficient manufacturing process with a phage cocktail. The associated clinical trial was also met with significant challenges. Thus, PhagoBurn also provided a major understanding of the challenges in the design of clinical trials for human phage therapy, and other potential limitations that may occur with the manufacturing and administration of a phage treatment [91]. Table 2 lists the more recent phage therapy clinical trials that have been registered in ClinicalTrials.gov.

**Figure 3:**
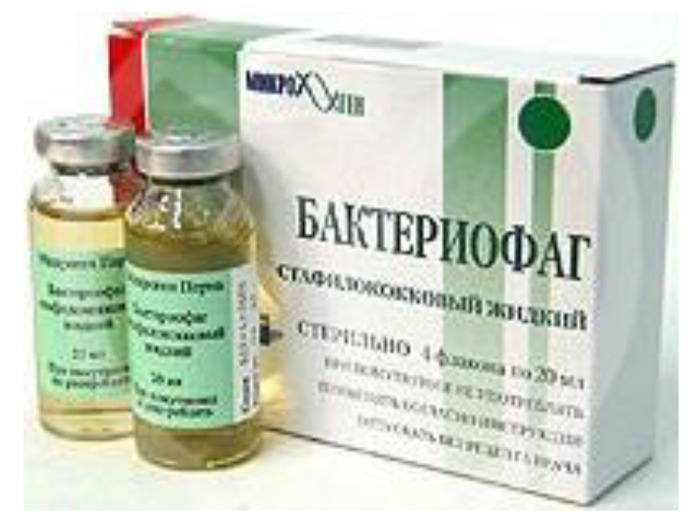
Sextaphage pharmaceutical product from microgen. Image reproduced with permission form reference [103].

As mentioned above, phage therapeutic products are currently on the market in certain parts of the world. Sextaphage is one such commercial pharmaceutical phage composition from the Russian company Microgen (Figure 3) [101]. This phage therapeutic contains phages against six specific pathogens, with the intent of treating urinary tract infections in pregnant women. Pregnant women are prone to urinary tract infections; however, very limited antibiotics have been proven safe for the developing fetus [102]. Microgen offers multiple other phage products for other infections. 

A recent notable successful clinical attempt of phage therapy was reported from scientists and physicians at the University of California San Diego School of Medicine, who had worked alongside collaborators from the U.S. Navy Medical Research Center-Biological Defense Research Directorate. As the first attempt in the United States, they successfully utilized intravenous phage therapy to treat a patient with a severe systemic infection caused by multidrug-resistant organisms. Due to the utilization of a specially curated phage therapeutic on a compassionate care level, this patient, a professor in the Department of Psychiatry at UC San Diego School of Medicine, was saved from an end-stage comatose condition [87]. The phages used in that case were isolated from various environmental samples. The UC San Diego case attracted significant publicity leading to the first case of clinical trial authorized in North America for intravenous phage therapy to be carried out at UC San Diego. 

In 2016, Paul Turner and colleagues reported isolating a phage that could restore antibiotic sensitivity in multidrug-resistant *Pseudomonas aeruginosa* [104]. This phage was later used to treat a patient with a longstanding aortic graft infection that did not respond to multiple surgical interventions and aggressive antibiotic therapy with a single application of phage [105]. The same team recently reported treating two cystic fibrosis patients with antibiotic-resistant infections. Aside from the wild type phage isolated from natural sources, genetically engineered phages have also been reported for phage therapy. A young patient with cystic fibrosis and bilateral lung transplantation who had developed a *Mycobacterium abscessus* infection was reportedly treated with a phage cocktail of genetically engineered lytic phages that were administered intravenously [106]. Genetically modified phages can circumvent some of the limitations of wild type phage (e.g., narrow host range); however, more indepth investigations are needed to assess the possibility of reversion under the niche selective pressures in the body. Finally, phage-derived proteins have been used as therapeutics [107]. The current review is mainly focused on whole phage virions and the reader is referred to the review article by Roach et al. for more information on using phage-derived biomolecules for therapy [108]. 

## 4. Phage-Host Evolutionary Dynamics and Diversification

Bacteria-phage interactions have remained central to the evolution and ecology of microbial communities, as they alter the competition of bacterial species and result in the evolution of new mutants/variants [109]. Phages and their bacterial hosts represent a predator-prey system. Hundreds of new predators (phages) are born each time a prey (bacterial cell) is eaten. Under selective pressure from phage predation, bacteria develop resistance to phages, and consequently, the bacteriophage counter-adapts and evolves to counter this resistance [110,111,112]. As a result of this co-evolution, phages have acquired counter bacterial defense mechanisms, including anti-CRISPRs [113]. It is partially due to this co-evolution that effective phage infection is not always observed regardless of phage adsorption [114].

Most mechanisms of phage resistance differ from that of antibiotic resistance [73,115]. In addition, as bacterial populations evolve to resist phage predation via random mutations, the predator phage will also shadow the bacterial community and mutate to combat the resistant bacteria, consequently increasing genomic diversity of both host and phage communities [110].

Due to differences in the mutational supply of both the bacteriophage and corresponding bacterial host and levels of resource supply for bacterial resistant mutations and phage infectivity mutations, there is a further difference in the strength of selection, population divergence, trajectory, and adaptation patterns [88,116]. Moreover, lytic phages typically have larger population sizes and shorter generation time in comparison to their corresponding bacterial host, which impacts the evolutionary race between bacteria and phage, leading to a higher diversification of the phage community compared to its host [117]. The strategic exploitation of multiple phages in the form of a therapeutic phage cocktail (and thus multiple selective pressures) has the potential to limit the bacteria’s ability to evolve resistance against a single phage species [118].

As the co-evolutionary arms race of phage and their corresponding host bacteria progresses, bacteria mutants resistant to phage predation will evolve. The nature of these mutations is believed to differ in vitro and in vivo and even in different niches in the body and for different phage-host pairs. What is common in all systems is the degree of diversification of the host community in response to phage predation, leading to a range of phenotypes that behave differently from the parental strain in terms of fitness [14,119]. In fact, the difference in behavior between the phage-resistant bacterial mutants and the parental strain was utilized to design phage therapy cocktails that lead an antibiotic-resistant bacterial population to regain antibiotic susceptibility [104].

Competition between the phage-resistant bacterial mutants and the entire niche microbiota for colonization and resources, nutrient availability, as well as the spatial heterogeneity of the niche microenvironment, could all affect the co-evolution of the phage-host system in a phage therapy case, leading to an outcome different from that predicted based on observations in a typical lab culture [120,121,122,123]. There are currently very few investigations on phage-host co-evolution in the gut environment. One of the few notable recent attempts is the work from the Institute Pasteur that reports phages evolving to infect different hosts in the gut [124]. It is important to study if the phenotypic and genetic diversity within the gut microbiota, and if the overall community environment structure could be maintained during the co-evolutionary processes underlying the interactions between bacteria and therapeutic lytic phages.

## 5. Bacteriophages and the Microbiota

### 5.1. Introduction to the Microbiota

The human body harbors a rich and complex community of microbial cells. This plethora of cells come from all three domains of life (archaea, bacteria, eukarya), with the number of bacteria in the body approximately the same order as that of eukaryotic-human cells, and phage outnumbering the bacterial population by a factor of 10 [125,126]. A symbiotic relationship exists between the human gut microbiome and the human host, the disturbance of which could lead to the initiation or progression of many inflammatory diseases of the gut, including Crohn’s disease [127], ulcerative colitis [5], and inflammatory bowel disease [128], among others. In addition, recent research has associated the gut microbiota with non-intestinal diseases such as rheumatoid arthritis [6], diabetes [129], obesity [130], certain forms of cancer [7], various neurological degenerative diseases [131], and various mental disorders [8]. Furthermore, evidence has been found linking the use of a wide range of drugs to alterations in the human gut microbiome, which could, in turn, impact the body’s susceptibility towards chronic and degenerative diseases [132,133,134,135].

### 5.2. Bacteriophages as Part of the Human Microbiota

Bacteriophages are naturally present within the human gut, oral and nasal cavity, genital tract, and eye, in collection with other bacteria, fungi, protozoa, and fungi. Most bacteriophages naturally present in the human body are temperate phages and many thus bacteria in our microbiome harbor prophages. Induction of this prophage under different conditions can lead to disturbance of the balance of the microbiome and dysbiosis (imbalance between the organisms present in our natural microflora) [136,137], necessitating further mechanistic investigations on the environmental triggers that induce temperate phage in the gut. Such induction can be a byproduct of diet, lifestyle, or other therapies such as chemotherapy. It can be deduced that controlled induction of the prophages in our commensal bacteria can be used as an approach to control the human microbiome in the case of perturbations of dysbiosis, and could thus be regarded as the next generation of phage therapy. Doing so, however, requires more knowledge about the triggers for prophage induction, a topic on which the current body of knowledge is at best incomplete. Another factor to consider is the interaction of the lytic phages used for therapy with the commensal phages (or prophages) in the microbiota. Specifically, induction of prophages as a direct or indirect result of lytic phage action could result in disturbance to the microbiota and thus further complication of the landscape in which therapeutic phage is expected to function.

### 5.3. Microbiome Therapy with Bacteriophages

Microbiome therapy aims to modulate the human microbiome as a therapeutic strategy to combat many of the chronic or degenerative diseases believed to be associated with dysbiosis in the gut microbiota or other niches in the body [138]. Specifically, due to the natural ability of bacteriophages for highly specific bacteria targeting, the exploitation of lytic or temperate phages as tools for microbiome therapy has the potential to manipulate and engineer the niche microbiota to achieve desirable effects for healthy balanced microflora [139,140]. This could be achieved using lytic phage for selective killing of certain species that have overgrown, leading to dysbiosis, or by selective induction of the temperate phage residing in commensal bacteria. The former, although more close to practice, suffers from the same bottlenecks as standard phage therapy, and the latter, although an attractive notion, is hampered by our very limited understanding of factors than can (selectively) trigger the lytic cycle for a temperate phage. Besides, the latter form of phage therapy requires the selective induction of temperate phage, a topic that has remained relatively unexplored to date.

### 5.4. Possible Interactions of Therapeutic Phage and Niche Microbiota

Despite the presence of commensal bacteriophages, naturally present within the human microbiota, the introduction of therapeutic bacteriophage to the microbiota will still act as an external agent [141]. The effect of lytic phage predation on densely colonized, nutrient-rich environments such as the human oral cavity [142] is different from the gastrointestinal environment, which although densely colonized, is affected strongly by nutrient limitations and thus promotes intense competition and cooperation among species [143].

In a recent report, researchers from the Wyss Institute at Harvard report that lytic phage simultaneously coexists and knocks down its host in the gut in a gnotobiotic mouse model colonized with human microbiota [144]. In addition, they show that phage predation can modulate the gut metabolome and claim that the effect on the metabolome is a result of non-susceptible bacteria co-colonizing species in the gut within a community of commensal bacteria colonizing the gut through cascading effects [144]. This work highlights the importance of the interactions between the commensal bacteria when a colonizing species is under phage predation and the potential impact of gut phages on the mammalian host with implications for both phage and microbiome therapies. 

Another investigation that highlights the impact of phage predation on non-targeted species is detailed in a report from Institute Pasteur, which investigates the possibility of phage co-evolution in the gut to evolve the ability to infect non-host bacteria [124]. In this report, Debarbieux et al. study a tripartite network consisting of a virulent bacteriophage, its bacterial host, and a phage-insensitive bacterial strain in the murine gut. They observed that single amino acid substitutions and an unusual homologous intragenomic recombination event within the genome of the bacteriophage enabled it to infect the insensitive strain in the mouse gut [124]. An intermediate bacterial host isolated from the murine microbiota was claimed to mediate bacteriophage adaptation.

It is important to note that the limited reports published on the effect of phage predation on the microbiota are mainly limited to the gut microbiome and do not seem to agree of the actual effect. Where certain groups claim phages induce minimal changes in phylogenetic compositions [145], others showed that phage predation results in compositional changes in the murine gut microbiota [146]. The inconsistency in reports can be partially attributed to the differences in models used (gnotobiotic versus different microbiota models) as well as the phage-host systems under study. Certain phages do not persist for more than a few hours in the gut environment, whereas others can persist for weeks or even months [147]. The ability of phage to persist in the gut environment can be attributed to its ability to infect and produce progeny in a predominately stationary phase host community. Furthermore, external factors may impact the successful administration and persistence of therapeutic phages within the gut microbiome, specifically, the external physical and chemical factors, such as acidity and presence of various ions. The utilization of certain therapeutic phages in acidic gastrointestinal environment may significantly reduce phage stability and phage titer [148,149,150]. In a fasting human, the median gastric pH is 1.7, in comparison to a pH over 6 in certain regions of the digestive tract [151,152]. To overcome this concern regarding the decreased stability from acidity, an antacid could be simultaneously consumed upon the administration of oral phage therapy, or the therapeutic phage is encapsulated in protective matrices [153,154,155].

Another possible contributing factor to the indirect effect of lytic phage predation on the niche microbiome is that immune response by the human host could be triggered as bacteriophages do have the potential to cause rapid and massive bacterial lysis, subsequently releasing components from the cell wall and phage proteins into the bloodstream [156]. Hence, the potential of an immune response that may induce the production of antibodies against phage action is of concern [141,157], and calls for more indepth studies on phage-immune interaction as well as effective dosing of phage therapeutics.

Currently, for acquiring regulatory approval it is important to prove the administered therapeutic phage (1) does not have the ability to integrate its genome into the genome of the target bacteria or any of the components of the niche microbiome setting off further indirect effects within the microbiome, (2) will not indirectly affect the microbiome through selective pressure on various non-target commensal bacteria or the target infectious agents, leading to development of phage-resistant mutants which can impact the balance surrounding beneficial bacteria in the niche, as well as the effects of antagonistic phage co-evolution (the reciprocal evolution of host resistance and parasite infectivity) on the gut microbiota. To obtain regulatory approvals, therefore, outstanding questions remain to be answered: does external phage therapy affect the microbiome? Specifically, does it affect non-target commensals? Do phage-resistant mutants emerge, and what is their impact on the microbiome? The literature in this field is scarce and contradictory, and despite impressive recent attempts, there remains a desperate need for more indepth investigations from the scientific community.

## 6. Conclusions

Phage therapy has the potential to cure drug-resistance pathogenic bacteria as well as patients that are intolerant to antibiotics. However, it is critical to further investigate and understand the detailed mechanistic interaction of bacteriophage as a therapeutic agent with the human microbiota, and specifically, any possible perturbations towards the niche microbiota as a result of the strong selective pressure exerted by lytic phage on their host communities. More mechanistic investigations that shed light on the nature of host-phage dynamics in the context of the niche microbiota will effectively illuminate and push forward the process to obtain regulatory approval for phage therapy in Western medicine.

## Figures and Tables

**Table 1:** Classification and Basic Properties of Bacteriophages [21].

Symmetry	Nucleic Acid	Order and Families	Genera	Members	Particulars
Binary (tailed)	DNA, ds, L	*Caudovirales*	15	4950	
		*Myoviridae*	6	1243	Tail contractile
		*Siphoviridae*	6	3011	Tail long, noncontractile
		*Podoviridae*	3	696	Tail short
Cubic	DNA, ss, C	*Microviridae*	4	40	
	ds, C, T	*Corticoviridae*	1	3	Complex capsid, lipids
	ds, L	*Tectiviridae*	1	18	Internal lipoprotein vesicle
	RNA, ss, L	*Leviviridae*	2	39	
	ds, L, S	*Cystoviridae*	1	1	Envelope, lipids
Helical	DNA, ss, C	*Inoviridae*	2	57	Filaments or rods
	ds, L	*Lipothrixviridae*	1	6	Envelope, lipids
	ds, L	*Rudiviridae*	1	2	Resembles TMV
Pleomorphic	DNA, ds, C, T	*Plasmaviridae*	1	6	Envelope, lipids, no capsid
	ds, C, T	*Fuselloviridae*	1	8	Spindle-shaped, no capsid

C: circular; L: linear; S: segmented; T: superhelical; ss: single-stranded; ds: double-stranded.

**Table 2:** Recent Phage Therapy Clinical Trials.

Trial Title	Condition/Infection	Intervention	Status	Country	Ref.
Standard Treatment Associated with Phage Therapy Versus Placebo for Diabetic Foot Ulcers Infected by *S. aureus*	Diabetic Foot, Staphylococcal Infections	PhagoPied: Topical anti-Staphylococcus bacteriophage therapy	Not Yet Recruiting	France	[92]
Individual Patient Expanded Access for AB-SA01, an Investigational Anti- *S. aureus* Bacteriophage Therapeutic	MDR *Staphylococcus aureus* infections	AB-SA01 (3- phage cocktail)	In Progress	USA	[93]
Individual Patient Expanded Access for AB-PA01, an Investigational Anti-*Pseudomonas aeruginosa* Bacteriophage Therapeutic	*Pseudomonas aeruginosa* infections (incl. MDR stains)	AB-PA01 (4-phage cocktail)	In Progress	USA	[94]
Safety and Efficacy of EcoActive on Intestinal Adherent Invasive *E. coli* in Patients With Inactive Crohn’s Disease	Crohn’s Disease	EcoActive (collection of bacteriophages)	Recruiting	USA	[95]
Analysis of changes in inflammatory markers in patients treated with bacterial viruses	Wide-range, non-healing postoperative wounds or bone, upper respiratory tract, genital or urinary tract infections whom extensive antibiotic therapy failed	oral, rectal and/or topical bacteriophage lysates/purified phage formulations/phage cocktails	Completed	Poland	[96]
Evaluation of Phage Therapy for the Treatment of Escherichia coli and Pseudomonas aeruginosa Wound infections in Burned Patients	Wound infection	PhagoBurn: *E. coli* phages cocktail (15-phage cocktail), *aeruginosa* Phages cocktail (13-phage cocktail)	Completed	Belgium, France, Switzerland	[91]
Bacteriophage Effects on *Pseudomonas aeruginosa*	Cystic Fibrosis (CF)	Mucophages (10-phage cocktail)	Completed	France	[97]
Therapeutic bacteriophage preparation in chronic otits due to antibiotic-resistant *Pseudomonas aeruginosa*	Antibiotic-resistant *Pseudomonas aeruginosa* in chronic otitis	Biophage-PA	Completed	United Kingdom	[98]
Antibacterial Treatment against Diarrhea in Oral Rehydration Solution	ETEC and EPEC Diarrhea	Oral T4 phage cocktail	Completed	Bangladesh	[81]
Bacteriophages for treating Urinary Tract Infections in Patients Undergoing Transurethral Resection of the Prostate	Urinary Tract Infections (UTI)	Intravesical instillation- PYO phage	Completed	Georgia	[99]
A Prospective, Randomized, Double-Blind Controlled Study of WPP-201 for the Safety and Efficacy of Treatment of Venous Leg Ulcers	Venous Leg Ulcers	WPP-201 (8-phage cocktail)	Completed	USA	[100]
